# Cross-Talks between the Cardiovascular Disease-Sarcopenia-Osteoporosis Triad and Magnesium in Humans

**DOI:** 10.3390/ijms22169102

**Published:** 2021-08-23

**Authors:** Marie-Eva Pickering

**Affiliations:** Rheumatology Department, CHU Gabriel Montpied, 63000 Clermont-Ferrand, France; mepickering@chu-clermontferrand.fr

**Keywords:** osteoporosis, sarcopenia, vascular calcification

## Abstract

Magnesium (Mg) is a pivotal and very complex component of healthy aging in the cardiovascular-muscle-bone triad. Low Mg levels and low Mg intake are common in the general aging population and are associated with poorer outcomes than higher levels, including vascular calcification, endothelial dysfunction, osteoporosis, or muscle dysfunction/sarcopenia. While Mg supplementation appears to reverse these processes and benefit the triad, more randomized clinical trials are needed. These will allow improvement of preventive and curative strategies and propose guidelines regarding the pharmaceutical forms and the dosages and durations of treatment in order to optimize and adapt Mg prescription for healthy aging and for older vulnerable persons with comorbidities.

## 1. Introduction

Aging is a major risk factor for pathologies affecting the cardiovascular, muscle, and bone domains, and is associated with an increased incidence of many chronic diseases, such as muscle loss and sarcopenia, vascular, metabolic conditions, and osteoporosis. Loss of bone (osteoporosis), of muscle (sarcopenia), impaired cardiovascular systems (including calcification and arterial stiffness), and aging share common mechanisms [1]. These chronic age-related diseases are intertwined and have molecular, physiological, and pathological links and share risk factors and clinical implications [2]. A number of interventional and therapeutic approaches are recommended for each of these pathologies, and guidelines/recommendations have been published by respective societies. Post-menopausal osteoporosis affects millions of people worldwide and this public health problem will increase in the coming decades [3,4]. It is characterized by bone loss and deterioration of micro-architecture of the bone. Beyond first-time drug treatment with bisphosphonates, non-pharmacological approaches with exercise, nutritional, and mineral supplements are advised [5]. Osteoporosis often coexists with sarcopenia and cardiovascular disease [2]. Sarcopenia, a concomitant loss of muscle mass and muscle function, leads to an impaired quality of life and an increased mortality [6,7]. Dietary approaches are important and may play a role in its pathophysiology and prevention [8,9,10]. Osteoporosis and cardiovascular disease are also linked [11,12], as patients with a low bone mineral density or an increased bone turn-over have a greater risk of cardiovascular morbidity and mortality, and dietary approaches are also recommended [13,14]. Within this cardiovascular disease-sarcopenia-osteoporosis triad, it is obvious today that apart from pharmacological treatment and combined with it, non-pharmacological approaches, including mineral intake, play a pivotal role to slow down the ailments of aging [5,15].

Magnesium (Mg), an intracellular cation, Mg^2+^, is one of the key micronutrients in the body. It is linked to the metabolism of calcium and potassium and has numerous structural and regulatory functions. It is involved in many enzymatic reactions and although an adapted diet should provide Mg daily needs, supplementation is frequent and Mg is commonly bought over the counter to fight against fatigue or stress [16]. Aging is very often associated with Mg deficiency and the Food and Drug Administration (FDA) recommends a daily oral Mg intake of around 400 mg for males and 310 mg for females [17]. Aging and Mg deficit are both associated with excessive production of oxygen-derived free radicals and low-grade inflammation [18,19,20], and are probably involved in the development of age-related chronic diseases.

This review aimed at studying Mg influences on each component of the triad and at discussing how Mg could be a useful polyvalent tool in slowing down concomitant pathological processes on cardiovascular, muscle, and bone systems. After discussing the cardiovascular disease-sarcopenia-osteoporosis triad, this review evaluated Mg effects in evidence-based medicine in order to identify possible bridges within this triad and how Mg may be a common denominator. It also focused on the existing gaps concerning the prescription and use of oral Mg in order to obtain a concomitant beneficial effect on the components of the triad.

## 2. The Cardiovascular Disease-Sarcopenia-Osteoporosis Triad

**Figure 1:**
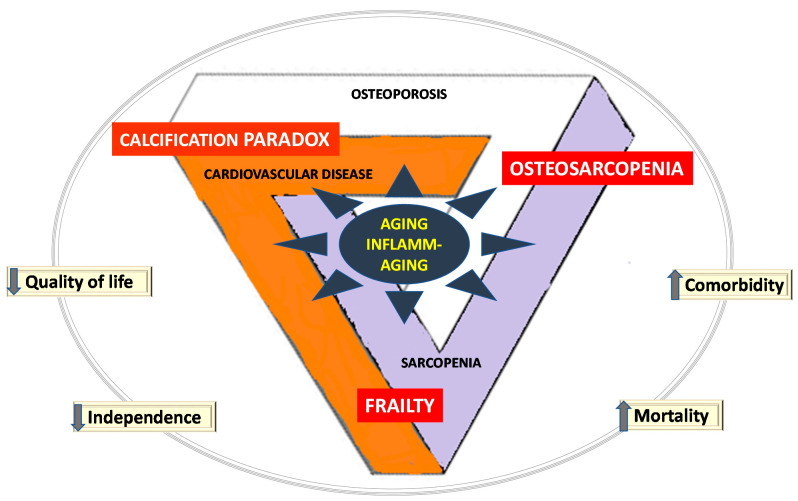
Interactions and issues in the cardiovascular-muscle-bone triad. Concepts have emerged in aging, with inflamm-aging [20] at the root of the development of age-related chronic diseases. Aging patients may develop osteosarcopenia [21], a combination of osteoporosis and sarcopenia; they may develop diminished calcification in osteoporosis with increased arterial calcification, namely the calcification paradox [22]; they may also develop frailty, sarcopenia, and cardiovascular disease [23]. These interactions participate in diminishing (down arrows) quality of life and independence and increasing (up arrows) comorbidity and mortality in aging persons [1].

Cardiovascular disease, sarcopenia, and osteoporosis occur in the course of aging with a large impact on quality of life, mobility, morbidity, independence, and mortality [2] (Figure 1).

### 2.1. Cardiovascular Disease

Increased life expectancy is accompanied by a rising prevalence of age-related cardiovascular diseases, including hypertension, heart failure, atherosclerosis, endothelial dysfunction, aortic calcification, myocardial infarction, and stroke [24]. Hypertension is complex and the World Health Organization [25] reports that, worldwide, one in four males and one in five females, totaling over a billion people, have hypertension, and that the figures will double by 2050. Aging and hypertension are two independent risk factors of cardiovascular disease, but have similar mechanisms in triggering cellular response, molecular pathways, and gene expression. Oxidative stress, production of free radicals, neuroendocrine, and genetic changes are involved. Cardiac changes with aging proceed, however, at a slow pace, but involve all components of the heart and vasculature. They manifest themselves clinically when alterations of cardiac physiology and function have reached a pathological state [26]. Dysfunction of the endothelium, with its reduced production/action of relaxing mediators, is pivotal in the pathogenesis of cardiovascular diseases, especially in hypertension. A healthy endothelium is indeed more than just a mechanical barrier, and modulates vascular tone by the synthesis and release of relaxing, vasodilating (prostaglandins, nitric oxide (NO)…), and contracting factors [27]. Endothelial dysfunction is one of the earliest indicators of cardiovascular dysfunction. Flow-mediated dilation (FMD) is the most widely used method to study endothelial function, but is operator-dependent, and new circulating markers of endothelial dysfunction, endothelial microparticles, endocan, and endoglin, are being explored [28]. Arterial stiffness may also be predictive of cardiovascular disease and pulse wave velocity (PWV) is considered the gold standard to assess arterial stiffness [29]. Aortic valve calcification is the most common valvular disease with high morbidity and mortality rates, and research still bears on factors modulating the osteogenic differentiation of human aortic valve interstitial cells [22]. In the context of the COVID-19 pandemic, pre-existing cardiovascular disease seems to be linked with worse outcomes and increased risk of death in patients with COVID-19 [30].

### 2.2. Sarcopenia

Sarcopenia is a skeletal muscle disorder characterized by loss of muscle mass and function. It is associated with physical disability, falls, fractures, morbidity and mortality, poor quality of life, depression, and hospitalization [31,32]. Recommendations for health care professionals [5,6] aim at an early detection and diagnosis in order to prevent or delay adverse outcomes. The prevalence of sarcopenia is reported to be up to 29% for older community-dwelling adults, up to 33% for individuals living in long-term care institutions, and up to 50% for patients ≥80 years old [33]. Muscle mass and muscle strength decrease with age and muscle dysfunction/sarcopenia is multifactorial. A number of age-related factors are present in sarcopenic older adults, such as denervated motor units, hormonal changes, inflammation oxidative stress, decline in physical activity, or malnutrition [34]. Recent publications underline how COVID-19 infection may aggravate sarcopenia because of the reduced physical activity and inadequate nutrient intake caused by social isolation [35]. A growing body of evidence on mitochondrial impairment in sarcopenia has been provided by both animal and human studies, as mitochondrial dysfunction is determinant in age-related loss of skeletal muscle mass and strength [36]. To date, research focuses on the impact of nutritional and pharmacological mitochondria-targeted interventions [36] and on the development of biomarkers, use of drug and non-drug approaches with nutrition and resistance exercise, and high grade clinical trials [37]. 

### 2.3. Osteoporosis

Osteoporosis is a multifactorial systemic skeletal disease characterized by increased bone resorption, low bone mineral density, and structural deterioration in bone microarchitecture [4]. With the rapid increase in average life expectancy worldwide, it has become an important public health issue as the incidence rate increases with age and over 70% of those over age 80 are affected [3], suggesting a dramatic increase of osteoporotic fractures in coming decades. As recently reported [3], the figures are impressive: over 50% of postmenopausal white women will have an osteoporotic-related fracture, and 75% of those with acute vertebral fracture may suffer from persistent back pain. Only 33% of senior women who have a hip fracture will be able to return to independence. This bone loss increases the risk of fragile and compression vertebral fractures, leading to pain, reduced mobility, and a vicious circle of secondary fracture, impaired quality of life, and diminished autonomy, increased morbidity, and mortality [38]. Post-menopausal osteoporosis is often called the “silent thief”, as the weakening of all bones is usually painless and progresses slowly, over many years, without any clinical symptoms until a fracture occurs and pain chronicization takes place. Bone integrity and pain outcomes are inconsistent and osteoporosis diagnosis relies on established criteria and dual-energy X-ray absorptiometry (DXA) measurement [4]. Acute sudden severe pain often signals the presence of a fracture that will require a multidisciplinary approach in order to restore physical function, improve overall conditioning, bring pain relief, and prevent future fractures. Osteoporotic fractures have a medical cost, but also an economic and societal one, and many fractures could be avoided through early diagnosis. Medications approved for osteoporosis by the US Food and Drug Administration (FDA) and the European Medicines Agency (EMA) are antiresorptive drugs (bisphosphonates, denosumab, selective estrogen receptor modulators (raloxifene), anabolic agents derived from parathyroid hormone (teriparatide), and romosozumab, an anti-sclerostin antibody. These therapies are often taken on a 6–10 years term, and are effective in reducing the risk of fractures [39]. Osteoporosis management has been particularly difficult in the COVID-19 pandemic with the disruption to the provision of health care globally, including requirements for social distancing [40].

## 3. Cross Talks in the Cardiovascular Disease-Sarcopenia-Osteoporosis Triad

Deep and conceptual interactions exist between the components of the triad [2] (Figure 1). Cardiovascular disease and osteoporosis share common pathogenetic mechanisms: patients with osteoporosis have higher levels of calcification than those with normal bone mineral density, and vascular calcification is related to a higher risk of fracture [11,12,41,42]. There is a continuum of disturbed and deviant mechanisms, termed “calcification paradoxes”, where aortic valve interstitial cells re-differentiate into an osteoblast-like phenotype, the pivotal cellular mechanism of aortic valve calcification [22]. Vascular calcification can impact skeletal muscle function, perfusion, and oxygen delivery to the muscle [34]; it is associated with five-year decline in sarcopenia (evaluated by hand grip test) in older women [43]. Cardiac dysfunction is also associated with sarcopenia; prevalence of sarcopenia in chronic heart failure amounts to up to 20%, and may progress into cardiac cachexia [44]. 

Osteosarcopenia has been described as a phenotype when osteoporosis and sarcopenia are present; it is a multifactorial condition linking bone and muscle, including genetics, age, obesity, and inflammation [21,45]. Frailty, a dysfunction of homeostatic mechanisms with reduction of the physiological reserve, is also predicted by cardiovascular disease [46], osteoporosis [47], and by muscle wasting [44]. Furthermore, sarcopenia in combination with frailty doubles the mortality risk of each condition [48]. 

Chronic sterile low-grade inflammation has also emerged as a common denominator to age-related diseases, with the concept of inflamm-aging [49], a landmark of frailty. With age, a vicious circle of production of inflammatory mediators in response to chronic endogeneous and exogeneous stimuli is set in motion and leads to inflamm-aging and the development of comorbidities including cardiovascular disease, osteoporosis, and sarcopenia [49]. 

## 4. Magnesium and the Triad

**Figure 2:**
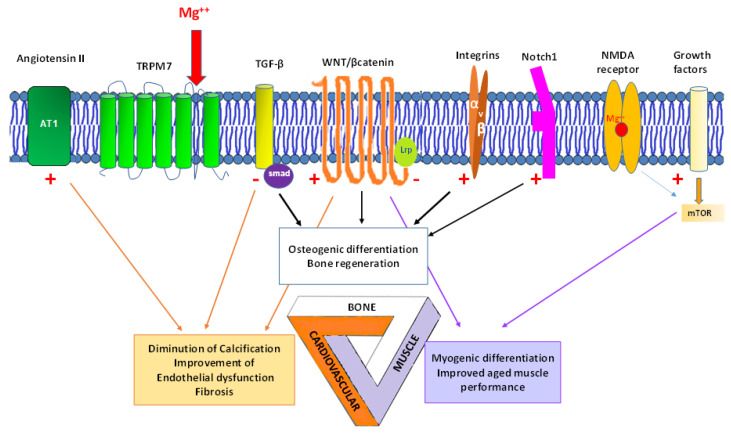
Magnesium supplementation (Mg^2+^)’s effect on the cardiovascular-muscle-bone triad. Mg activates (red +) and inhibits (red −) molecular ligands. Mg enters the cell via the TRPM7 receptor (and other channels). It is a physiological blocker of the NMDA receptor. At the bone level, regeneration and osteogenic differentiation are facilitated by the activation of Wnt/β-catenin [50], Integrins [51], Notch-1 [52], and the inhibition of TGF-β [53]. At the cardiovascular level, diminution of calcification, improved endothelial function, and fibrosis are facilitated by the activation of angiotensin II receptor [54], Wnt/β-catenin [55], and the inhibition of TGF-β [56]. At the muscle level, myogenic differentiation and improved performance are facilitated by the activation of mTor [57] and the inhibition of Wnt/β-catenin [55].

Mg has multiple regulatory functions in the cardiovascular-muscle-bone triad (Figure 2).

### 4.1. Magnesium and Cardiovascular Function 

A number of prospective studies have analyzed the link between Mg and cardiovascular disease, vascular calcification, vascular smooth muscle tone, endothelial function, hypertension, mortality, or prevention [58,59,60,61,62].

In vitro studies suggest the protective role of Mg on vascular calcification. Mg influences vascular calcification by impairing hydroxyapatite crystal growth [63]. It has also been shown in vitro to inhibit Wnt/β-catenin activity and to reverse the osteogenic transformation of vascular smooth muscle cells [55]. Low Mg levels are associated with vascular calcification and Mg enters the vascular smooth muscle cells through the Transient Receptor Potential Melastatin 7 (TRPM7) channel (also via other channels), a channel also used by other ions like calcium or zinc. Angiotensin II, the vasoconstricting peptide hormone, stimulates TRPM7 activity, and prevents vascular calcification via the influx of Mg, the inhibition of the canonical Wnt/β-catenin signalling pathway and the induction of ERK1/2 MAPKinase [54].

In humans, studies have explored the correlation between Mg blood levels and mortality [58], cardiac disease [64], vascular calcification [59], endothelial function [62], stroke [65], and revascularization [66].

The relationship between Mg and cardiovascular disease mortality is complex. Low serum Mg levels have been shown to be associated with increased cardiovascular disease (CVD) and all-cause mortality in the general population [64]. A recent meta-analysis of prospective studies (including over 1 million participants) was conducted to examine the association of total, supplemental, and dietary Mg intakes with the risk of CVD mortality risk, and showed no association with a lower CVD mortality risk. The study, however, showed that consumption of Mg from dietary sources may be beneficial in reducing all-cause and cancer mortality [67]. The role of dietary Mg intake in reducing CVD mortality has been described with a dose-response relationship irrespective of gender [68] and only found among women [69]. Drinking water Mg level is associated with the risk of CHD mortality (RR = 0.83, 95% CI = 0.69–0.98) [70]. Cardiovascular mortality has been studied in patients with chronic kidney disease, and the relationship between Mg blood levels and mortality is not as clear [71]. A five-year prospective cohort study showed that lower-serum Mg was associated with a higher risk of cardiovascular and all-cause mortality in the peritoneal dialysis population, especially among female patients [72]. In patients with chronic kidney disease, both hypo and hypermagnesemia were associated with higher all-cause mortality, but not with an increased risk of CKD progression [73]. Hypermagnesemia, but not hypomagnesemia, at the time of hospital admission was associated with increased 1-year mortality among hospitalized patients, probably linked to latent chronic disease [74]. A recent meta-analysis showed that Mg concentration is inversely associated with all-cause mortality and cardiovascular mortality and events [75]. A score (Magnesium Depletion Score (MDS)) has been recently validated to predict the Risk of Systemic Inflammation and Cardiovascular Mortality among US Adults, and to identify individuals with Mg deficiency who may benefit from increased intake of Mg [76].

Cardiac adverse events are common among aging patients. The possible role of hypomagnesaemia as a cardiovascular risk factor may be explained by the development and amplification of atherosclerotic plaques and coronary spasm. Serum Mg in randomized patients with acute myocardial infarction is indeed lower compared with controls in some studies [77], but hypermagnesemia did not prevent the occurrence of cardiac adverse events [78] in a large population of stroke patients. Several prospective studies report an inverse association between elevated serum Mg level, elevated Mg intake, and cardiovascular disease [64].

Mg, like other electrolytes (potassium, calcium…), has an effect on the electrical conduction of the heart. Mg deficiency is associated with heart rhythm abnormalities [79] and atrial fibrillation [80], and Mg supplementation has been shown to be useful in atrial fibrillation [81], arrythmia [82], and torsade de pointes [83]. Oral Mg supplements also improved survival outcome in patients with congestive heart failure [84].

While iv MgSO4 in the immediate postinfarction period in a hundred patients is cardioprotective with a reduction of arrhythmias, pump dysfunction, and death [77], larger studies do not show this causal link. Treatment of acute stroke patients with Mg did not result in a reduction in the number or severity of cardiac-serious adverse events in the randomized FAST-MAG trial [78], on mortality outcomes in the MAGIC (6000 participants [85]), ISI 4 trial (58,000 patients) [86], RCTs, or in the US Registry for myocardial infarction [87] where Mg use was associated with increased mortality.

Following cardiac revascularization, the effect of MgSO4 was also studied in a meta-analysis on cardiovascular events, which showed that the total rate of cardiac arrhythmia was significantly lower in the group receiving MgSO4 than the group receiving the placebo. It showed also that Mg consumption would decrease ventricular (in 15 RCTs) and supraventricular arrhythmias (in 14 RCTs) compared with the placebo group [66].

Correlations have been described between low serum Mg and coronary artery calcification after multiple adjustments in a population at low risk of cardiovascular disease [65,88]. Mg dietary intake has been the focus of attention for a number of years, as inadequate intakes of Mg have been underlined for the last two decades [89]. Dietary Mg intake and improved cardiac health has been shown in the Framingham Offspring Study [90], with Mg intake >320 mg/day leading to a 34% lower risk of cardiovascular disease, but not in other studies [64]. Improved mortality [69] and lesser vascular calcification are related to Mg dietary supplementation [91]. An updating of adult Mg requirements has been recently suggested in 2021, as mean body weight has increased over the last ≥20 years [92]. High Mg intake levels are also associated with a lower risk of major cardiovascular disease (diabetes, hypertension, metabolic syndrome) and stroke [59].

Mg has also an impact on the endothelial function [62,93,94], which is itself linked to blood pressure changes. Oral Mg supplementation in randomized clinical trials (RCTs) resulted in a significant improvement of endothelium-dependent brachial artery flow-mediated vasodilation (a marker of endothelial function) in patients with comorbidities: in those with coronary artery disease [95], obesity [96], diabetes [97], in those on hemodialysis [98,99], or in hypertensive [80] individuals for durations of 6 months to 2 years.

Mg supplementation has shown heterogenous effects on hypertension, moderate in some studies [69,90], and a meta-analysis showed that 1–6 months of Mg supplementation resulted in a reduction in systolic blood pressure (4 mmHg) and in diastolic blood pressure (2 mmHg) [60]. With a dietary Mg range of 96–425 mg/day, a statistically significant inverse association with hypertension risk has been shown, with a 100 mg/day increment in Mg intake being associated with a 5% reduction in the risk of hypertension [69]. Intervention studies with dietary supplementations have used organic and inorganic forms of Mg, oxide, chloride, citrate, diglycine, aspartate, pidolate, citrate, lactate, and etc., with a range of 250 to 1800 mg per day, for 30 days up to 2 years depending on studies. The most recent review [60] proposed a classification of patients in order to overcome the conflicting results concerning Mg supplementation in hypertension. Oral Mg (≥240 mg/day) safely lowers blood pressure in uncontrolled hypertensive patients taking antihypertensive medications, while >600 mg/day Mg is required in untreated hypertensives. A supplementation of <600 mg/day may help to improve other risk factors without antihypertensive medication side effects [60].

Mg is ubiquitous in affecting vascular function and protecting against excitotoxicity mediated by NMDA receptors. Mg supplementation has been used for decades in clinics for eclampsia and neurological disorders [100], and is safe and inexpensive. However, the neuroprotective effect of Mg supplementation on stroke is still controversial and different clinical approaches and routes of administration are described, ranging from intravenous to more invasive Mg administration and combination therapies. Several prospective studies have reported an inverse association between Mg intake/supplementation (and calcium and phosphorus) and the risk of stroke [101,102]. Mg is suggested to act in a dose-dependent manner: for each 100 mg/day, the risk is reduced by 2% [65]. However, a recent study in 6411 interviewed participants (aged 45–79 years) free of stroke at baseline suggested that only dietary calcium intake, but not Mg, is associated with a lower risk of stroke in Chinese adults, particularly in men [103]. The observational nature of the studies and lack of RCTs is a limiting factor. Intravenous (iv) Mg supplementation (MgSO_4_) versus placebo has been studied in large clinical trials [104], like a large multicenter trial in 2589 patients that failed to demonstrate a real benefit [105], or a randomized phase three trial with prehospital initiation of Mg in 857 patients that did not improve disability outcomes at 90 days [106]. Mg supplementation for cerebral ischemia has been administered with more invasive techniques (in the lateral ventricle, combined with hypothermia therapy, or with intracarotid Mg supplementation and selective hypothermia). A recent paper showed that subintracisternal MgSO_4_ infusion improves clinical outcomes without complications in patients with poor-grade subarachnoid hemorrhage, with additional effects when combined with intravenous hydrogen therapy [107].

### 4.2. Magnesium and Muscle

Sarcopenia is defined as a combination of low muscle mass with low muscle function, with diminished muscle strength, muscle quality, and quantity [6,108,109]. It affects 10% to 50% of older persons and contributes to physical disability, impaired quality of life, and mortality. Inflamm-aging, dysregulation of immunosenescence, and sedentary lifestyles all participate in muscle wasting. Muscle mass loss increases over the years, reaching up to 50% in persons >80 years old. Nutrition may influence the development of sarcopenia [8], and older persons are often at nutritional risk [110].

Intramuscular Mg pool (27% of total body Mg) diminishes with age, and this influences muscle health since Mg, as with calcium, is involved in muscle contraction/relaxation. Animal studies show that Mg has an impact on muscle performance [6,109] and that a number of signalling pathways are involved, with muscle stem cells at the heart of muscle regenerative capacity. A recent study in animals showed that Mg, via the mTor receptor, the protein kinase mechanistic target of rapamycin, facilitates myogenic differentiation, improves aged muscle performance, and conserves muscle mass and strength [57]. A mild Mg deficiency may change the expression of genes critical for muscle physiology, including energy metabolism and muscle regeneration, even before the emergence of muscle dysfunction [111].

In humans, a number of publications have explored the links between Mg blood levels/dietary intake with muscle and sarcopenia [8,9,112,113,114,115,116,117,118,119]. Among seven cross-sectional studies [9,113,114,115,116,119,120], five underline a low Mg dietary intake in patients with sarcopenia [113,114,117,119,120]. Significant lower intakes were found in those with sarcopenia compared those with no sarcopenia in 227 community-dwelling older adults, but this difference was not reflected in a difference in serum Mg levels, probably because Mg is strictly regulated by the kidney, gastrointestinal tract, and bone [120]. Mg intake was 6% lower in 66 sarcopenia patients matched to 66 non-sarcopenic persons [114]. In a cross-sectional study issued from the UK BioBank, the highest tertiles of intake for Mg were all associated with the lowest likelihood of sarcopenia [113]. The EPIC Norfolk cohort study [117] showed significant positive trends in fat-free mass measures across Mg dietary intake for men and women, but this was not reflected in serum Mg levels. In a recent study focusing on frailty and Short Physical Performance Battery to measure physical function, non-frail participants “at risk of malnourishment” showed a significantly lower intake for Mg (392.2 ± 83.9 mg/day compared with those “not at risk of malnutrition”), 451.3 ± 111.6 mg/day (*p* = 0.016) [119]. An inverse correlation between dietary Mg with greater muscle mass and muscle power was also observed in the UK Biobank, EPIC-Norfolk, Tasmanian cohorts, and the TwinsUK registry [11,115,117,118]. One Italian study showed a significant positive association of serum Mg concentration with muscle performance in 1453 community residents [112]. Finally, only one trial studied Mg supplementation in 139 healthy older women (not sarcopenic) [116], showing that 300 mg Mg oxide supplementation for 12 weeks (with weekly exercise) resulted in better muscle and physical performance. A significant improvement of total Short Physical Performance Battery scores, including five-time chair-stand, tandem balance evaluation, and 4 m walking speed was observed, but this concomitant physical activity prevented conclusions bearing on the causal role of Mg supplementation. In the light of these observations, there is a need for RCTs to elucidate the potential benefits of mineral intake to prevent and/or treat sarcopenia and support healthy aging.

### 4.3. Magnesium and Bone

Mg in bone represents 60% of total-body Mg and plays a role in bone turn-over [5]. Studies carried out in animals or in vitro have shown a significant association between Mg and bone mineral density [121,122,123], with a direct or indirect effect of Mg [124,125,126,127]. The regulatory mechanisms led by Mg and its transporters across the membrane, in mineralization, and in osteoblast generation are, however, not fully understood. Mg ions promote osteogenic differentiation of mesenchymal stem cells [128], but high concentrations of extracellular Mg ions may inhibit mineralization [129]. Solute carriers across the membrane [129], parathyroid hormone, cytokines, TGFβ [53,56], Wnt/β-catenin [50], integrins, MAP kinases [51], and Akt-mTor [130] signalling pathways are all known to play a role in osteogenic differentiation. Mg probably uses these pathways to modulate bone ossification, but the fine tuning of this modulation with physiological Mg levels or supplemented Mg may suggest different mechanisms. For example, Wnt signaling is central in osteogenesis and in determining the transformation of stem cells into mature osteoblasts. Activation of the canonical Wnt signalling pathway results in nuclear translocation of β-catenin, hence regulating target gene expression [130], and Mg has been shown, in vitro, to induce an osteogenic effect in the bone marrow space by activating this pathway [50]. In animals, severe Mg deficiency compromises systemic bone mineral density and aggravates inflammatory bone resorption [131]. However, Mg supplementation has been shown to increase mesenchymal stem cell proliferation in a dose-dependent manner and to promote osteogenic differentiation and mineralization with activation of Notch1 signalling, but with no increase in the canonical Wnt/β-catenin pathway [52].

In humans, a few reviews have updated knowledge on Mg and bone health [5,132,133]. A recent narrative review [133] identified 28 studies since 2009 on the link between Mg and bone in adults, mostly postmenopausal women, considering blood Mg values, Mg intake, and Mg supplementation. Lower blood Mg values are globally associated with osteoporosis, and 30–40% of the analyzed studies reported patients with hypomagnesemia. Seven prospective/observational studies have focused on the link between Mg blood levels and bone health, as a primary outcome, in postmenopausal osteoporosis and/or in those >60 years old [123,134,135,136,137,138,139]. Compared with controls or osteopenia persons, osteoporotic women had globally lower serum Mg concentrations, lower than the reference range or remaining within the reference range with or without statistical difference. The different reference ranges for serum Mg may introduce a methodological bias between studies, especially on the minimal value of the range, i.e., 1.9–2.5 mg/dL [136], 1.6–2.4 mg/dL [123]. A link between magnesemia and fracture risk has been shown in the literature [137,138]. Lower-than-recommended daily Mg intake (265 mg/day in many studies) is linked to a lower bone mineral density, and a higher fracture risk is observed [133].

Eight studies, prospective [135,137,140,141,142] and cross sectional [9,143,144], have studied the relationship between Mg dietary intake and bone health in persons with postmenopausal osteoporosis and/or those >60 years old. Evaluation was carried out with a questionnaire, food diary, or Food Frequency Questionnaire. These showed a lower-than-average dietary Mg intake and a positive association between Mg intake and bone mineral density, or specific markers of bone resorption like type-I collagen C-Telopeptide (CTx) [140]. The different reference ranges for the dietary Mg intake requirement (265 mg/day [140], cut off values (<206 mg/day) [141], or use of quintiles [9] for analysis limit comparisons between studies. Studies performed over the last decade have shown that 20% of the general population consume less Mg than recommended, and have a lower bone mineral density and a higher fracture risk [9,133,137,142]. Supplementation studies in persons with postmenopausal osteoporosis and/or those >60 years old are limited. The case-control (n = 31 vs. 23 controls [145]) (n = 26 vs. 7 controls [146]), retrospective (n = 53 (6 only with Mg) [147]), and only one randomized (n = 20 supplemented, 10 unsupplemented with no placebo [148]) studies showed an improvement of bone-turnover markers, of bone mineral density, and prevention of fractures [141,145]. Dietary supplementation has used Mg citrate (1830 mg/day for 1 month [148]), or oxide (250–750–250 mg/day for 2 years [145] or 1200 mg/day [146] for 6–12 months). The paucity of RCTs calls for more trials in older persons, concomitantly taking into account Mg blood levels, baseline Mg intake, standardized supplementation, duration of supplementation, and menopausal status with a double blind and placebo approach.

## 5. Prospects of Mg in the Cardiovascular Disease-Sarcopenia-Osteoporosis Triad

Osteoporosis, cardiovascular disease, and the development of muscle aging and degeneration develop with aging and have consequences on the risk of falls, fracture, and quality of life [23,149]. Microenvironmental factors involving protein ligands like Notch, Wnt, mTor, myokine, myostatin, fibroblast growth factor, and minerals like Mg are all at play in each component of the triad. Mg is a key element in these three domains, as shown by fundamental research and cross-sectional, case-control, and intervention studies (Figure 2). Molecular, animal, and clinical studies show correlations between Mg and the components of the triad. After Mg entry in the cell via the TRPM7 receptor (and other channels), it may switch on or off the Wnt/β-catenin signalling pathway, leading respectively to improved osteogenic differentiation and diminished vascular calcification. It also disrupts the TGFβ mediated-smad pathway improving fibrosis and activates mTor, leading to myogenic differentiation and halting muscle mass loss, with other interactions and molecular cascades that are still being explored. Overall, the message arising from the literature is that circulating Mg, dietary Mg intake, or Mg supplementation in healthy persons, postmenopausal women, older persons, patients with chronic comorbidities like chronic kidney disease, diabetes, hypertension, or coronary heart disease converges towards a beneficial effect on bone, the cardiovascular system, and muscle. Preventive measurement of Mg levels has also been underlined [150]. Mg appears, therefore, as an interesting molecule for preserving healthy aging and the integrity of the triad. The number of RCTs is, however, limited with oral Mg (one [116] in sarcopenia, one [148] in osteoporosis, a few in endothelial function [62], with the bulk of RCTs being in hypertension [60]), and with iv Mg SO4 in acute situations like stroke and infarction. Beneficial tendencies of Mg described in RCTs confirm the positive findings in non-RCTs and observational studies, but a number of gaps are present in the literature.

First, a large heterogeneity of the methodologies used in the different trials is observed. Different populations, pathologies, missing information on severity of the patients’s disease, different oral Mg chemical forms, or different settings are reported. A recent review [151] observed that eight chemical Mg forms have been studied head-to-head for bioavailability, and concluded that second-generation organic salts (citrate, pidolate, gluconate) are more bioavailable than first-generation inorganic salts (carbonate, chlorure, and oxide). It also stresses that third generation organic salts (bisglycinate, glycerophosphate) are more bioavailable than the other two generations. However, no study compared head-to-head Mg pharmaceutical forms in any of the conditions of the triad. This point is particularly important for prescribing Mg: satisfactory bioavailability of Mg salts does not allow us to deduce that this would lead to increased efficacy on bone, cardiovascular, or muscle domains. Another gap is that besides beneficial effects, Mg adverse events, although usually minor, are poorly reported in the studies and in real life, while there is a large use of Mg, prescribed or obtained over the counter or on the internet. Likewise, Mg blood levels are not always reported unless when specifically focused as an end point of the study. The occurrence of hypermagnesemia needs also to be also considered, especially in the context of long-term supplementation for diseases affecting the triad. High Mg concentration has been shown in vitro to inhibit the mineralization process and to modulate gene expression of mesenchymal stem cells during osteogenic differentiation. Involvement of Mg transporter SLC41A1 [103] and of Mg substitution for calcium in the hydroxyapatite structure [152] has been suggested.

Studies also vary in Mg daily dosages and the duration of treatment, making it difficult to identify a reference salt and an optimal duration of Mg supplementation. So far, no universal dosage has been defined. Clinical trials aimed at defining an optimal dosage of Mg or testing different generations of Mg salts at recommended FDA dosages are necessary to determine whether differences in efficacy occur depending on the generation used. Such an approach would help therapeutic strategy not only in osteoporosis, cardiovascular disease, and muscle impairment, but also when comorbidities like bone fractures, stress, chronic pain, psychological symptoms, and impaired quality of life are present, as is often the case in aging persons [16]. Such studies could lead to recommendations for using Mg preventively, all the more since Mg deficiency, because of poor diet intake or other pathologies, is associated with aging. More information is also needed for a curative approach in patients, as in diabetes, obesity, or in chronic kidney disease.

The use of Mg could also be valuable for other comorbidities of the triad, like pain. As regards the inflamm-aging concept, within the cardiovascular disease-sarcopenia-osteoporosis triad and in other concomitant age-related diseases, a number of comorbidities, including other rheumatic diseases like osteoarthritis [32], are painful, and pain has a dramatic impact on the quality of life and independence of older persons. For example, the combined presence of several painful pathologies like osteoporosis, diabetic neuropathy, sarcopenia, and osteoarthritis traps an older patient in a vicious pain circle. Muscle weakness, axial kyphosis, contraction in paravertebral muscles to maintain posture, loss of stature, and bone loss all contribute to pain, disability, fracture occurrence, and further risk of new fractures and loss of autonomy. Patients may also have orthopedic surgery after a fracture and experience post-surgical pain, another risk factor for the development of chronic and neuropathic pain [153], with peripheral and central mechanisms of pain at play. The use of Mg in pain relies on the fact that Mg is the physiological blocker of the N-Methyl-D-aspartate (NMDA) receptor, and controls its hyperexcitability in pain chronicization and in learning, cognitive, and emotional processes [151]. The molecular cascades described in pain are also thought to impact sleep, anxiety, and fatigue [151,154]. A recent review [151] reported a modest effect of Mg in chronic pain but also stressed that there is to date in the literature no RCT evaluating the benefits of Mg supplementation on pain occurring in osteoporosis, rheumatic disease, or sarcopenia. Finally, Mg has been suggested as a supportive treatment of COVID-19, especially in critically ill patients [155].

## 6. Perspectives for Future Trials on the Triad

To better understand the role of Mg on the cardiovascular-muscle-bone triad, the first step would be to identify the concomitant links between the three components of the triad in a longitudinal study, with a homogeneous population, and then apply Mg supplementation on the set model. The choice of the population as a mode of entry into the triad is determinant for reliable findings and subsequent extrapolation. Cardiovascular disease exploration allows a number of objective measures (blood pressure, FMD, PWV, arterial calcification…), but is an umbrella term for a large array of pathologies that may be present at different degrees in the same person, some linked to high mortality. Sarcopenia is also heterogeneous, but benefits today from consensual evaluation criteria for follow-ups [7]. The entry within the triad via osteoporosis/post-menopausal osteoporosis allows objective bone evaluation (DXA) and accessible quantification at baseline and at follow-ups (bone turner markers); in addition, endpoints including cardiovascular and muscle performance parameters could be evaluated. A chronic disease like osteoporosis slowly progresses over years and would allow for the identification of the chronological trajectories of the three components of the triad over 3 to 4 years. Concerning Mg supplementation, a RCT in osteoporotic patients may be carried out in cross-over mode or in two or three parallel groups (requiring more participants), versus placebo or a second formula, with a primary endpoint (bone resorption marker like CTx, FMD, muscle mass, muscle function…), chosen according to the recruitment facilities, the needed number of persons, and the setting. The choice of the Mg formula, dosage, and duration of treatment relies on positive findings discussed in the literature. As mentionned, there is no face-to-face study of different Mg formulae, no systematic description of Mg adverse events in the studies, and no recommended, efficient formula. According to the literature, it is reasonable at least to replenish Mg depleted stores with oral Mg, as the aging population has a lower-than-recommended intake and often hypomagnesemia, iv MgSO4 applying for acute rather than chronic situations. The studies with oral Mg show a large range of dosages, formulae, and combinations. According to Mg recommendations in blood pressure and data on bone and sarcopenia [92,145], a possible protocol could be 300 to 750 mg/day elemental Mg for a duration of a few months to one year, including in the trial a second group taking a different formula/dose/increasing dose and/or duration, with surveillance of adverse events. Such a combination of a longitudinal study and a Mg supplementation RCT could allow us to identify how Mg may modulate in real life age-related chronic diseases for healthier aging.

## 7. Conclusions

Mg is a pivotal and very complex component of healthy aging in the cardiovascular-muscle-bone triad. Low Mg levels and low Mg intake are common in aging, and are associated with poorer outcomes than higher levels, including vascular calcification, endothelial dysfunction, osteoporosis, or muscle impairment/sarcopenia. While Mg supplementation appears to reverse these processes and improve better health prospects in the triad, more RCTs are needed. These will aid in improving preventive and curative strategies, proposing guidelines regarding the pharmaceutical forms, the dosages and durations of treatment, in order to optimize and adapt Mg prescription for healthy aging and for older vulnerable persons with comorbidities.

## Data Availability

Not applicable.
